# Sclerodermia and Raynaud's Disease

**Published:** 1909-09-25

**Authors:** 


### Medicine.

### SCLERODERMIA AND RAYNAUD'S DISEASE.

Neither sclerodermia nor Raynaud's disease is common, and therefore the fact that the two conditions have been observed together in the same individual again and again would seem to indicate that, dissimilar though they may appear to be at first sight, they have some essential common factor in their pathology. This raises several interesting questions, not the least of which is that deficient activity of the thyroid gland has been suggested as one such common factor, in which case the administration of thyroid extract in suitable doses, along with such local or other treatment as each individual case may seem to require, should be beneficial both in sclerodermia and in Raynaud's disease, whether these occur together or separately.

The following is a case in point:—A lady aged fifty-five sought advice for certain nervous and cutaneous troubles affecting both her body and her extremities. Beyond the fact that the father was a drunkard who died young, there seemed to be nothing of importance in the family history. The patient herself had had smallpox at four. At eleven she began to menstruate, and developed marked symptoms of a functional nervous nature at the time. With the appearance of the menses she was seized with an intense fear of the sight of blood; the same evening her mouth was twisted to the left side; liquids regurgitated through her nose, so that they could only be swallowed when the head was thrown right back; the voice became nasal and the patient could not articulate words. These symptoms persisted for two months, and then disappeared again as abruptly as they had developed.

She was married at nineteen, and she had an only daughter. The first husband died of acute phthisis; at thirty she married again, but had no family by her second husband, who also died of consumption.

The patient had always suffered more or less from gastric pains and discomfort, especially after meals; she had always been troubled with sleepiness also; but otherwise she had been fairly well, until at the age of thirty-two, having developed a rapid enlargement of her abdomen, she felt sure she was pregnant, and consulted a midwife and a physician. The diagnosis of pregnancy was at once changed for one of simple obesity with abdominal predominance. Almost immediately the patient became breathless easily, and declared that she could not mount stairs. Vaso-motor troubles became obvious. The face readily flushed; there was a sensation of intense internal heat, whilst any degree of either heat or cold externally became intolerable. The patient could only endure herself in the open air.

At thirty-nine she passed through a severe attack of typhoid fever lasting two months. To the nervous symptoms already detailed were now added some of a more mental character. Memory was affected, the patient became unduly sad, weeping for trivial causes; her ideas wandered, and she was bad at recollecting things. When she went out for walks she repeatedly lost herself in places with which she had formerly been quite familiar. At about forty-five years of age she added to the above symptoms those of violent headaches, severe cramp-like pains in her limbs, and shooting pains in her joints. The hands and feet swelled; when it was cold the extremities became blue and painful; when it was hot the patient felt them to be burning. The skin became thicker, the fingers were bulkier and more difficult to move.

These symptoms persisted; at fifty the menopause occurred. At fifty-five her condition was a combination of typical sclerodermia with Raynaud's disease, possibly with myxœdema also. A stout, short woman, she was able to walk only slowly and with difficulty. Her face was red, swollen-looking, and yet fixed and immobile, like that of a wax model or a mask; the cause of this fixity was the sclerosis of the subcutaneous tissues and skin. The mouth, when closed, was little more than a slit between the pinched lips, and it could only be opened very slightly to allow of the introduction of food. The hands were cold and of a dull red colour; the least coldness of the atmosphere brought out the typical second degree of Raynaud's disease in them. The fingers were stiff and cylindrical, hard to the touch, stiffened by cutaneous and subcutaneous sclerosis, and scarcely to be moved at all, either actively or passively. The sclerodermia was less marked in the forearm, though there were areas of it both here and on the legs and on the trunk.

All the fingers felt as if they were made of some firm, resistant, homogeneous substance. The skin could no longer be made to slide or move over the subjacent structures. The fingers themselves were cold, and there was a noticeable difference between their temperature and that of the rest of the hand. The line of demarcation between the cold and the warm zones was clearly defined, about the middle of both the back and the palm of each hand, but the regions liable to syncope and asphyxia did not coincide with those affected by sclerodermia. The syncopal areas came only to the middle of the hand, whilst the sclerodermia extended to the wrist.

The toes and feet were affected in a similar way to the fingers and hands, but to a less degree. The heart, lungs, kidneys, and digestive organs presented no obvious abnormality. The nervous system exhibited none of the ordinary signs of organic disease. The region of the thyroid gland felt quite empty. The palpating fingers came directly down upon the larynx and trachea without being able to feel either lateral lobe. It is true that, in the case in question, the administration of thyroid gland extract did not bring about any great diminution in the sclerodermia. This, however, does not prove that it would not prevent, or at least lessen, the rate of spread of the disease; and in future cases it is to be hoped that the earlier recognition of the nature of the lesions and the more prompt use of thyroid extract may prevent patients from advancing nearly so far in the wrong direction as was the case above.

